# Effect of Vaginal Microecological Alterations on Female Pelvic Organ Prolapse

**DOI:** 10.1007/s00192-024-05759-7

**Published:** 2024-03-15

**Authors:** Shaozhan Chen, Qiaomei Zheng, Limin Zhang, Lihong Chen, Jinhua Wang

**Affiliations:** 1 Department of Obstetrics and Gynecology, Fujian Key Laboratory of Precision Medicine for Cancer, the First Affiliated Hospital, Fujian Medical University, 20 Chazhong Road, Fuzhou, Fujian 350005 People’s Republic of China; 2 Department of Gynecology, National Regional Medical Center, Binhai Campus of the First Affiliated Hospital, Fujian Medical University, 999 Huashan Road, Fuzhou, Fujian 350212 People’s Republic of China

**Keywords:** Pelvic organ prolapse, Vaginal microbiome, Matrix metalloproteinase-3, Collagen I, Collagen III

## Abstract

**Introduction and Hypothesis:**

The objective was to investigate the correlation between endogenous vaginal microecological alterations and female pelvic organ prolapse (POP).

**Methods:**

Patients who underwent vaginal hysterectomy were retrospectively analyzed as the POP group (*n* = 30) and the non-POP group (*n* = 30). The vaginal microbial metabolites and enzyme levels were tested using the dry chemoenzymatic method. The mRNA and protein expression were tested using real-time quantitative PCR and immunohistochemistry. SPSS version 25.0 and GraphPad Prism 8.0 were performed for statistical analysis.

**Results:**

Compared with the non-POP group, the vaginal pH, H_2_O_2_ positivity and leukocyte esterase positivity were higher in patients with POP (all *p* < 0.05). Further analysis showed that patients with pelvic organ prolapse quantification (POP-Q) stage IV had higher rates of vaginal pH, H_2_O_2_ positivity and leukocyte esterase positivity than those with POP-Q stage III. Additionally, the mRNA expression of decorin (DCN), transforming growth factor beta 1 (TGF-β1), and matrix metalloproteinase-3 (MMP-3) in uterosacral ligament tissues were higher, whereas collagen I and III were lower. Similarly, the positive expression of MMP-3 in uterosacral ligament tissue was significantly upregulated in the POP group compared with the non-POP group (*p* = 0.035), whereas collagen I (*p* = 0.004) and collagen III (*p* = 0.019) in uterosacral ligament tissue were significantly downregulated in the POP group. Correlation analysis revealed that there was a significant correlation between vaginal microecology and collagen metabolism. In addition, MMP-3 correlated negatively with collagen I and collagen III (*p* = 0.002, *r* = −0.533; *p* = 0.002, *r* = −0.534 respectively), whereas collagen I correlated positively with collagen III (*p* = 0.001, *r* = 0.578).

**Conclusions:**

Vaginal microecological dysbiosis affects the occurrence of female POP, which could be considered a novel therapeutic option.

## Introduction

Pelvic organ prolapse (POP) is an extensively epidemic disease with common symptoms, including anterior and posterior vaginal wall bulge, uterine prolapse, and rectal bladder prolapse. According to statistics, the prevalence of POP in the USA is 6% to 46%, with a projected increase up to 50% by the year 2050 [1, 2]. A national multicenter cross-sectional survey in China suggests that 9.6% of adult female nationwide might have symptomatic POP [3]. Although there are a number of operative approaches to treating POP, the most commonly used method is hysterectomy combined with vaginal anterior and posterior wall repair. Nevertheless, unsatisfactory therapeutic outcomes and postoperative recurrence remain challenging issues, for which there are currently no predictors or effective preventive measures [4]. As reported, POP is a complex disease with multifactorial etiology that may be closely associated with aging, birth trauma, increased intra-abdominal pressure, obesity, ethnicity, and systemic comorbidities [5, 6]. However, the etiology and pathophysiology of this condition still remain unclear. Therefore, investigating its pathogenesis has become an urgent issue in current health care.

Vaginal microecology, the coexistence of various bacteria in the female vagina with the host in a dynamic equilibrium, plays a pivotal role in maintaining host health. In recent years, there has been increasing attention on the impact of vaginal microecological dysbiosis on gynecological diseases. Studies have shown that when the vaginal microecology is disrupted, it can lead to inflammation (e.g., bacterial vaginosis and endometriosis) and neoplasm [7, 8]. Most POP patients are elderly and may exhibit vaginal microecological and vaginal inflammatory manifestations owing to fluctuations in estrogen levels. It is hypothesized that the occurrence or progression of POP might be associated with vaginal microecological dysbiosis and inflammatory changes. However, there is a paucity of research on the correlation between vaginal microecology and POP. The current literature primarily focuses on the link between postmenopausal hypoestrogenism and POP [9, 10]. Further clinical research is warranted to investigate whether vaginal microecological dysbiosis interacts with hormones or directly contributes to the occurrence or progression of POP.

The pelvic floor connective tissue mainly consists of extracellular matrix (ECM), including collagen, elastin, and matrix metalloproteinase (MMP). Among them, collagen is the main component of ECM. The function of fibroblasts in POP may be associated with alterations in collagen levels [11, 12]. Abnormalities in the proportion of collagen components or structure and properties of collagen fibers within the connective tissue of the pelvic floor can result in decreased tissue toughness and elasticity, leading to weakened support for pelvic organs and ultimately contributing to the occurrence of POP [13]. Nevertheless, there is a lack of research on the relationship between vaginal microecology and collagen metabolism, and further evidence is needed to confirm the pathogenesis.

It can, thus, be hypothesized from the available data that vaginal microecological dysbiosis might be linked to POP, potentially contributing to its development or progression by altering collagen metabolism through related pathways. The current study is aimed at investigating the relationship between endogenous vaginal microecological alterations and POP, as well as exploring the potential mechanisms by which vaginal microecological dysbiosis may affect the occurrence of POP through facilitating collagen metabolism.

## Materials and Methods

### Patients and Samples

Uterosacral ligament tissues were obtained from 30 patients (median age: 63.50, range 51.25–69.75, pelvic organ prolapse quantification (POP-Q) stage III-IV, assessed by two qualified gynecologists), who underwent vaginal hysterectomy and met the inclusion criteria at the Department of Obstetrics and Gynecology of the First Affiliated Hospital of Fujian Medical University from December 2019 to December 2020. Normal uterosacral ligament tissues were obtained from 30 women without POP (median age: 59.50, range 52.00–64.25) as controls. We gathered participant information, including age, body mass index (BMI), number of deliveries, comorbidities such as diabetes and hypertension, as well as vaginal secretion indicators such as vaginal pH, hydrogen peroxide (H_2_O_2_), leukocyte esterase (LE), sialidase (SNA). Tissue samples from the uterosacral ligament were collected from both groups following vaginal hysterectomy. Informed consent was obtained from all participants prior to surgery. Ethical approval was supported by the Ethics Committee of the First Hospital of Fujian Medical University, Fuzhou, Fujian, China (approval number: 2019 [040]).

### Evaluation Parameters

Patients who had not undergone vaginal surgery or medication, douche, or topical or oral estrogen within 1 month prior to the collection of vaginal secretion samples were included in this study. The dry chemistry enzyme method (Shuosi vaginitis combined test kit, Suo-mei registration: 20,192,401,247) was utilized by adding four drops into designated wells and allowing them to settle for 5 min to detect the pH value, H_2_O_2_, LE, and SNA. The functional assessment of vaginal microecology was based on the relevant criteria outlined in the Clinical Practice of Vaginal Microecology Evaluation System, committee opinion [14]. Normal vaginal pH ranged from 3.8 to 4.5, whereas a pH level ≥ 4.6 was considered abnormal. A positive H_2_O_2_ concentration was defined as < 2 μmol/l, a positive LE concentration was ≥ 9 U/l, and a positive SNA concentration was ≥ 7 U/l.

### Experiment Methods

#### Real-Time Quantitative PCR (SYBR® Green qPCR Method)

The mRNA expression levels of decorin (DCN), transforming growth factor beta 1 (TGF-β1), matrix metalloproteinase-3 (MMP-3), collagen I, and collagen III were quantified by real-time quantitative PCR (qPCR). RNA was extracted from human uterosacral ligament tissues with POP and non-POP using Trizol reagent according to the manufacturer's protocol. qPCR amplification and lysis curves were confirmed at the end of the reaction to obtain Ct values. The relative quantification method (RQ = 2^−∆∆Ct^) was employed to determine expression differences of DCN, TGF-β1, MMP-3, collagen I, and collagen III between two groups. 18 s served as an internal reference with Ct = Ct_purpose_-Ct_control_. Three replicate wells were set up for each reaction (Table 1).

**Table 1:** Gene primer sequence

Primer		Sequence (5’ → 3’)
DCN	Forward	TCTCTGTAGTTGGATCAAGTGACT
	Reverse	TCTGAAGGTGGATGGCTGTAT
TGF-β1	Forward	TAAGAAGACGTTCACCAAGCCC
	Reverse	CTCCGATCACAAAGGCTGCAAA
MMP-3	Forward	ATGCTGTTTTTGAAGAATTTGGGTT
	Reverse	GTCACTTTCTTTGCATTTGGGTCA
Collagen I	Forward	GAGAGCATGACCGATGGATTC
	Reverse	CTTCTTGAGGTTGCCAGTCTG
Collagen III	Forward	TTGGATGCTATCAAGGTATTCTGT
	Reverse	GGAAGTTCAGGATTGCCGTAG
18 S	Forward	AGAAACGGCTACCACATCCA
	Reverse	CACCAGACTTGCCCTCCA

*DCN* decorin, *TGF-β1* transforming growth factor beta 1, *MMP-3* matrix metalloproteinase-3

#### Immunohistochemistry

The protein expression of MMP-3, collagen I and collagen III were assessed using immunohistochemistry (IHC). The specimens were fixed in 10% neutral formalin, embedded in paraffin, and sectioned at 4-μm intervals. The histological sections underwent heating at 70°C for 15 min, followed by xylene treatment to remove the paraffin, graded ethanol solutions, and distilled water for rehydration. Sections were boiled in 1 mol/l Tris–EDTA buffer to 95–98°C for antigen retrieval and placed at room temperature for at least 30 min. Antigen repair was conducted using a pressurized citrate thermal buffer with a pH of 6. Detection was carried out with proteintech IHC kits (KHC0109, KHC0205, KHC0264) following the manufacturer's instructions. Image analysis was performed on an OLYMPUS biomicroscope.

#### Criteria for the Interpretation of Immunohistochemistry Staining

A four-level grading method was employed to interpret the scoring criteria. The IHC-stained sections were scanned using the KF-PRO-005-EX digital section scanner and analyzed using K-Viewer software to sample the point range. Staining intensity and percentage of positive cells were observed at ×400 magnification in ten randomly selected fields of view, from which an immunoreactive score (IRS) was obtained. The IRS was calculated by multiplying the cell-staining intensity score with the percentage of positive cells score. Cell-staining intensity was graded on a scale of 0 to 3, where 0 represented no positive staining, 1 represented light yellow staining, 2 represented brownish-yellow staining, and 3 represented Tan staining. The percentage of positive cells was classified into five levels: 0 points for < 5%, 1 point for 5–25%, 2 points for 26–50%, 3 points for 51–75%, and 4 points for 76–100%.

#### Statistical Analysis

The software SPSS version 25.0 (SPSS, Chicago, IL, USA) and GraphPad Prism 8.0 were utilized for statistical analysis. Normally distributed data were expressed as mean ± standard deviation, whereas non-normally distributed data were expressed as median (P25 to P75). Continuous variables were compared using either Student’s *t* test or Mann–Whitney *U* test, whereas qualitative variables were compared using either the Chi-squared test or Fisher's exact test. Spearman's or Kendall rank correlation analysis was conducted to investigate the association between vaginal microecology and collagen metabolism. A *p* value < 0.05 was considered statistically significant.

## Results

### Correlational Analysis of the Vaginal Microecology and Pelvic Organ Prolapse

There were no significant differences in age, BMI, number of deliveries, diabetes mellitus or hypertension between the POP group and non-POP group (see Appendix Table 7 and Table 8). Compared with the non-POP group, the POP group exhibited significantly higher levels of vaginal PH, H_2_O_2_ positivity and LE positivity. However, there was no significant difference in salivary acid glycosidase positivity between the two groups (*p* = 0.754, Table 2). Further stratification of the POP group was performed, with 13 cases classified as POP-Q stage III and 17 cases classified as POP-Q stage IV, and results indicated that patients with POP-Q stage IV had higher rates of vaginal pH, H_2_O_2_ positivity, and LE positivity than those with POP-Q stage III (Table 3).

**Table 2:** Comparison of vaginal microecological function outcomes between two patient cohorts

Group	pH value, *n* (%)		H_2_O_2_ positivity, *n* (%)	LE positivity, *n* (%)	SNA positivity, *n* (%)
	≥ 4.6	3.8–4.5			
POP group (*n* = 30)	22 (73.3)	8 (26.7)	23 (76.7)	20 (66.7)	7 (23.3)
Non-POP group (*n* = 30)	14 (46.7)	16 (53.3)	14 (46.7)	11 (36.7)	6 (20.0)
Chi-squared	4.444		5.711	5.406	0.098
*p* value*	**0.035**		**0.017**	**0.020**	0.754

*POP-Q* pelvic organ prolapse quantification, *H*_*2*_*O*_*2*_ hydrogen peroxide, *LE* leukocyte esterase, *SNA* sialidase

*p values from Chi-squared test for unequal variances

Significant data (*p* < 0.05) are indicated in bold

**Table 3:** Comparison of vaginal microecological function outcomes between two patient cohorts

Group	PH value, *n* (%)		H_2_O_2_ positivity, *n* (%)	LE positivity, *n* (%)	SNA positivity, *n* (%)
	≥ 4.6	3.8–4.5			
POP-Q stage III (*n* = 13)	6 (46.2)	7 (53.8)	7 (53.8)	7 (53.8)	4 (30.8)
POP-Q stage IV (*n* = 17)	16 (94.1)	1 (5.9)	16 (94.1)	13 (76.5)	3 (17.6)
*p* value*	**0.009**		**0.025**	0.255	0.666

*POP-Q* pelvic organ prolapse quantification, *H*_*2*_*O*_*2*_ hydrogen peroxide, *LE* leukocyte esterase, *SNA* sialidase

*p values from Fisher's exact test for unequal variances

Significant data (*p* < 0.05) are indicated in bold

### The mRNA Expression Levels of DCN, TGF-β1, MMP-3, Collagen I, and Collagen III were Quantified by qPCR in the POP and Non-POP Groups

**Fig. 1:**
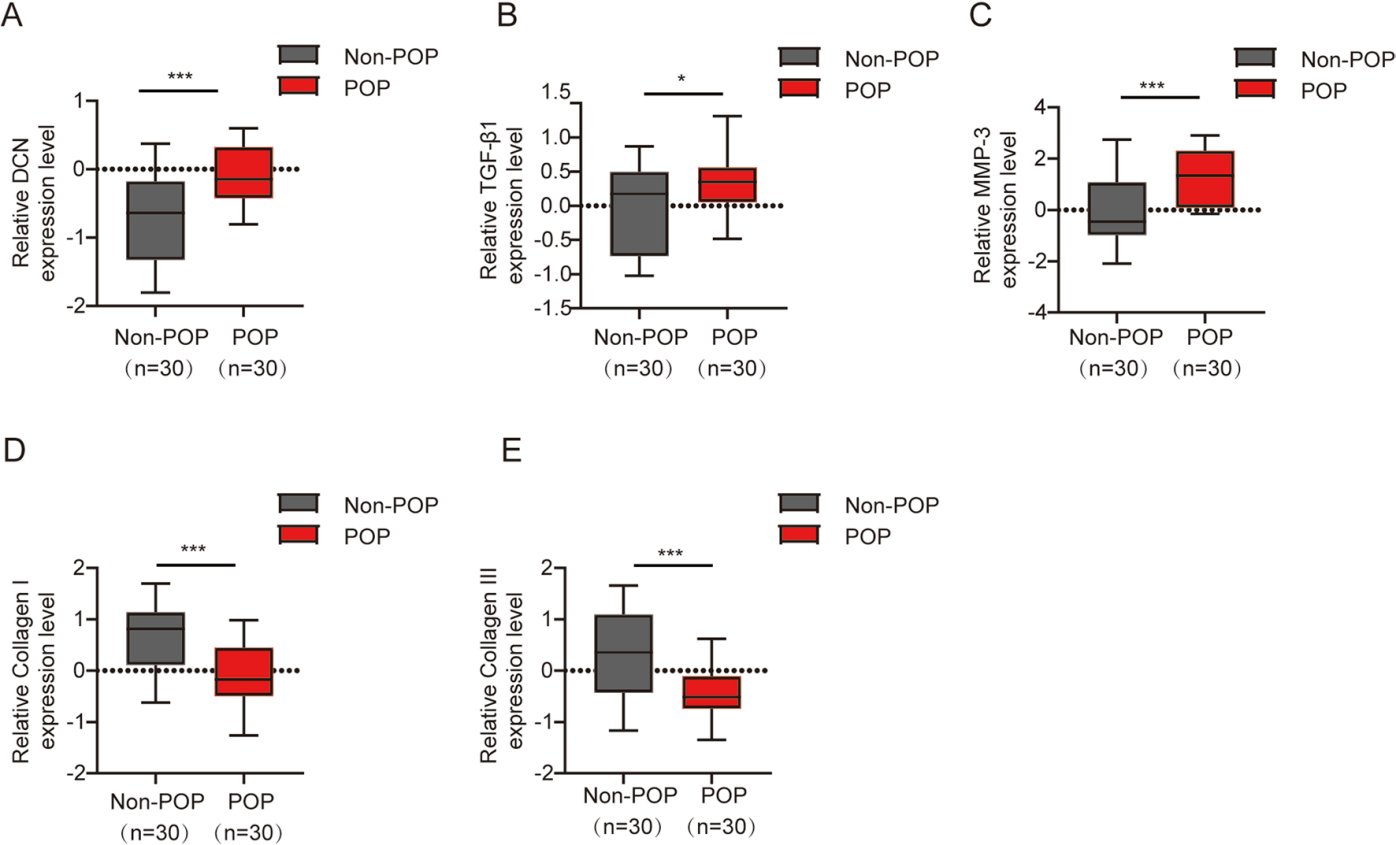
The mRNA expression levels of **A** decorin (*DCN*), **B** transforming growth factor beta 1 (*TGF-β1*), **C** matrix metalloproteinase-3 (*MMP-3*), **D** collagen I, and **E** collagen III in tissue samples from the uterosacral ligament were quantified using quantitative polymerase chain reaction in patients with pelvic organ prolapse (*POP*) and non-POP. Statistical analysis revealed significant differences between the two groups (*p < 0.05, ***p < 0.001)

The findings revealed that the mRNA expression levels of DCN, TGF-β1, and MMP-3 were significantly higher in the POP group than those in the non-POP group (Fig. 1A–C), Conversely, the mRNA expression levels of collagen I and III were markedly lower in the POP group than those in the non-POP group (Fig. 1D, E).

### Protein Expression of MMP-3, Collagen I, and Collagen III was Evaluated by IHC in the POP and Non-POP Groups

**Fig. 2:**
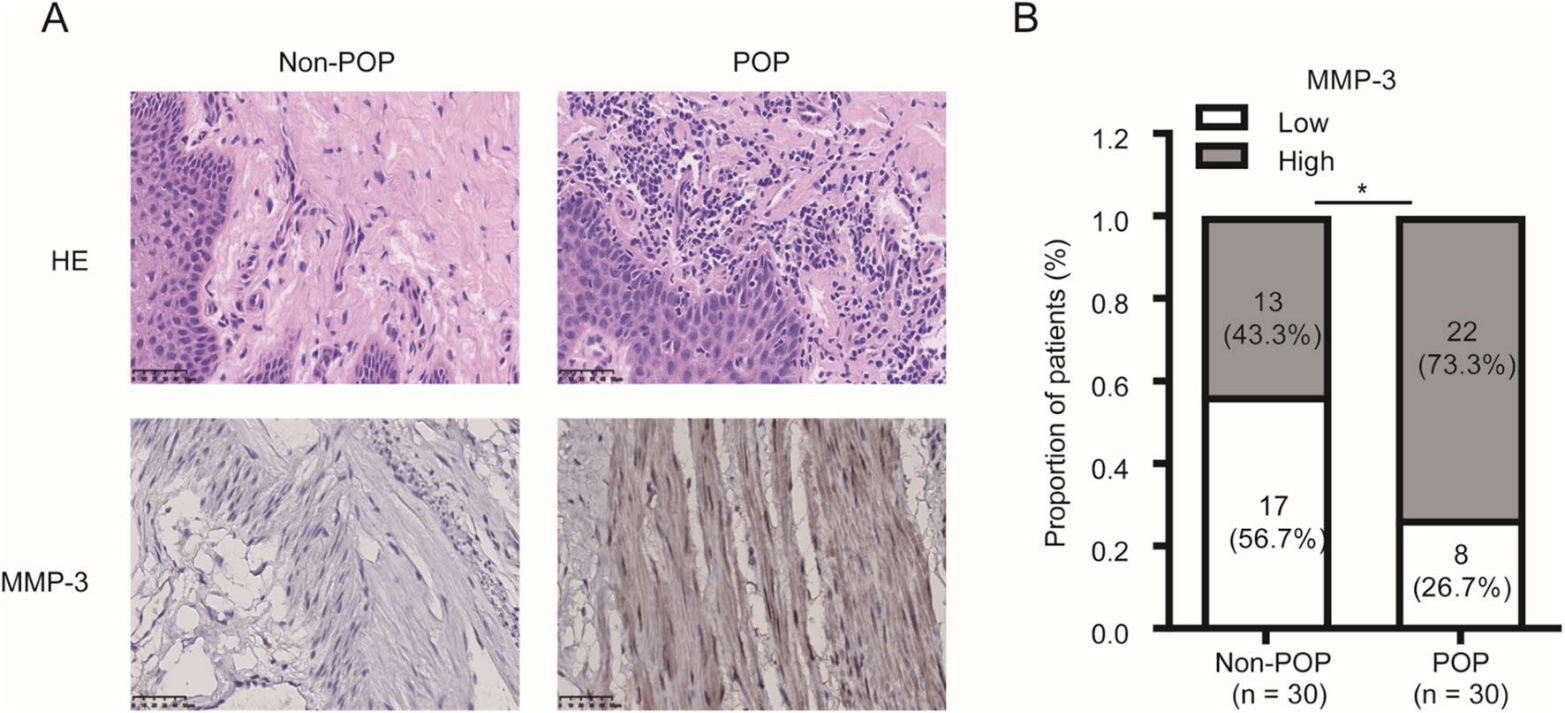
Matrix metalloproteinase-3 (*MMP-3*) is significantly upregulated in pelvic organ prolapse (*POP*). Tissue sections were subjected to **A** hematoxylin and eosin (*HE*) staining and **B** immunohistochemistry analysis for MMP-3 expression in both POP and non-POP tissues (bar, 50 μm). *p < 0.05

**Fig. 3:**
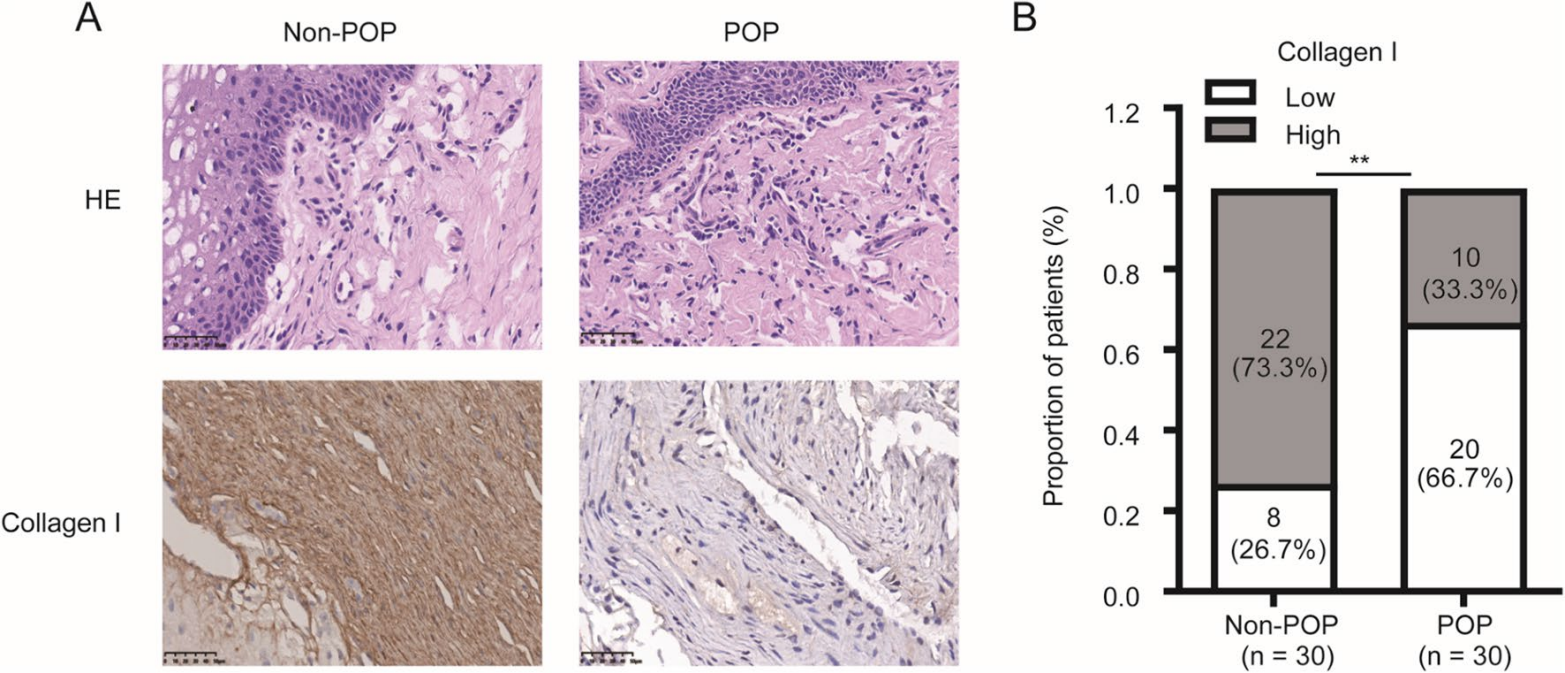
Collagen I expression is significantly lower in pelvic organ prolapse (*POP*) than in non-POP. Tissue samples were **A** stained with hematoxylin and eosin (*HE*) and **B** subjected to immunohistochemistry analysis for collagen I expression. (bar, 50 μm). **p < 0.01

**Fig. 4:**
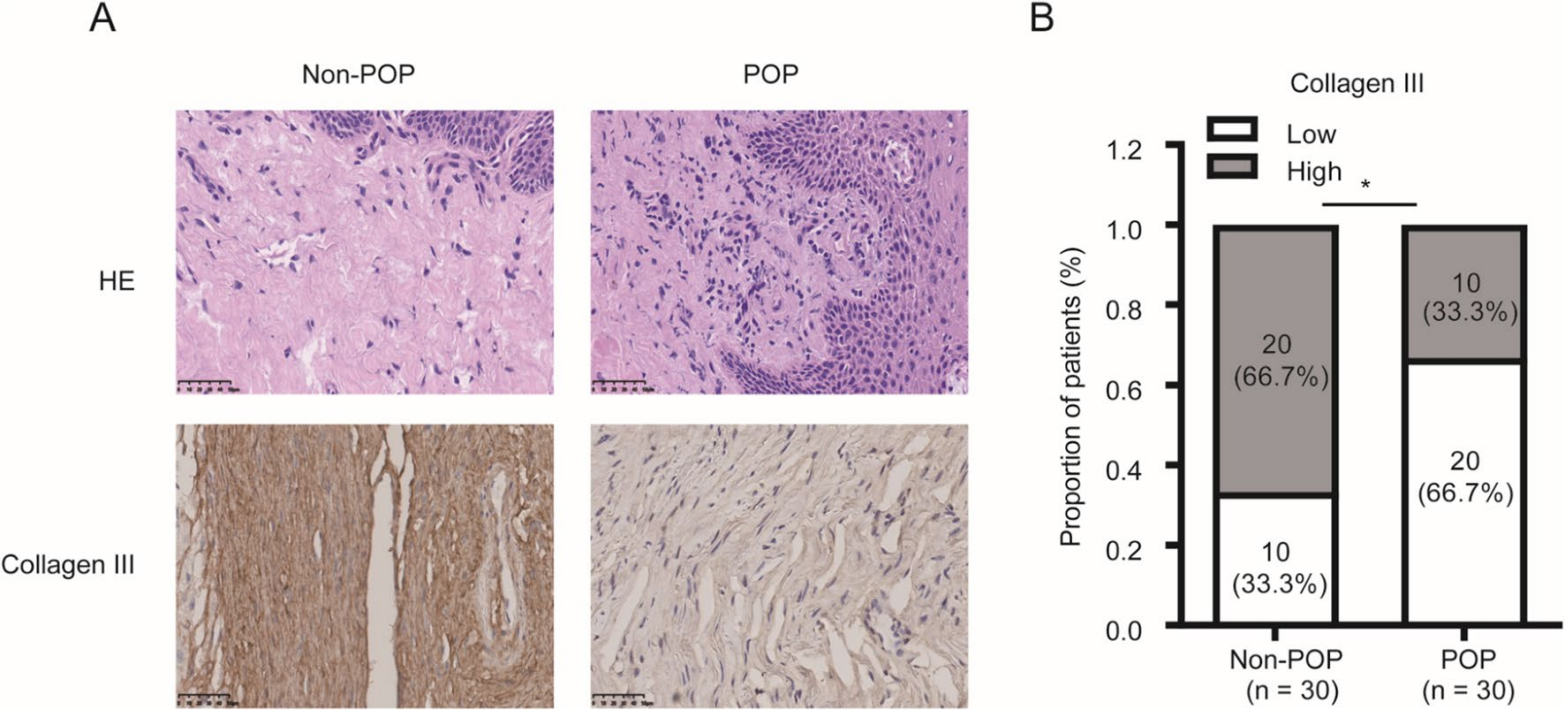
Collagen III expression is significantly lower in pelvic organ prolapse (*POP*) than in non-POP. Tissue samples were **A** stained with hematoxylin and eosin (*HE*) and **B** subjected to immunohistochemistry analysis for collagen III expression. (bar, 50 μm). *p < 0.05

The findings indicated that the positive expression of MMP-3 in uterosacral ligament tissue was significantly upregulated in the POP group compared with the non-POP group (*p* = 0.035, Fig. 2). However, the positive expression of collagen I and collagen III in uterosacral ligament tissue was significantly downregulated in the POP group compared with the non-POP group (*p* = 0.004, Fig. 3; *p* = 0.019, Fig. 4).

### Correlation Analysis between Vaginal Microecology and Collagen Metabolism in Patients with POP

There was a significant positive correlation between vaginal pH and MMP-3 expression (*p* = 0.033, *r* = 0.331). However, no significant correlation was observed between pH and collagen I or collagen III (all *p* > 0.05). H_2_O_2_ positivity demonstrated a significant positive correlation with MMP-3 (*p* = 0.013, *r* = 0.384), as well as a negative correlation with collagen I (*p* = 0.014, *r* = −0.381) and collagen III (*p* = 0.013, *r* = −0.395). LE positivity also showed a positive correlation with MMP-3 (*p* = 0.023, *r* = 0.352), whereas no significant correlations were observed between LE positivity and collagen I or collagen III (all *p* > 0.05).

**Fig. 5:**
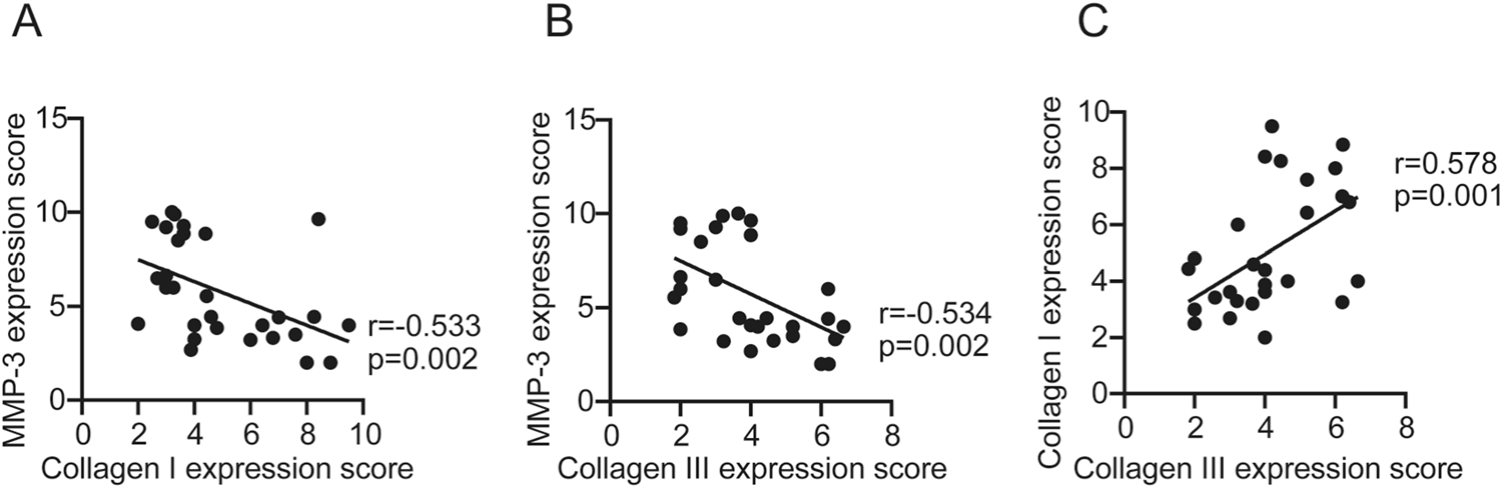
**A–C** Correlation of matrix metalloproteinase-3 (*MMP-3*), collagen I, and collagen III with clinical characteristics in patients with pelvic organ prolapse

The MMP-3 exhibited a negative correlation with both collagen I and collagen III (Fig. 5A. *p* = 0.002, *r* = −0.533; Fig. 5B; *p* = 0.002, *r *= −0.534), whereas collagen I demonstrated a positive correlation with collagen III (Fig. 5C. *p* = 0.001, *r *= 0.578) based on the results of IHC staining and interpretation criteria.

### Correlation of MMP-3, Collagen I, and Collagen III with Clinical Characteristics in Patients with POP

The protein expression levels of MMP-3, collagen I, and collagen III were analyzed in relation to clinical parameters (age, menopause, BMI, and POP-Q stage) of patients with POP (*n* = 30), using IHC staining results and interpretation criteria scores combined with clinicopathological tissue sample information.

Based on the IHC staining results and interpretation criteria, MMP-3 expression was classified as high expression (≥ 5 points) and low expression (< 5 points), with a median interpretation result of 5 points. The findings indicated that there were 15 patients with high expression of MMP-3 and 15 patients with low expression among 30 samples of POP tissue. Notably, elevated levels of MMP-3 were significantly associated with menopause (*p* = 0.021) and the severity of POP (*p* = 0.003, Table 4).

**Table 4:** Clinical relevance of matrix metalloproteinase-3 (*MMP-3*) in pelvic organ prolapse

Group	Age (years)	Menopause, *n* (%)	BMI (kg/m^2^)	POP-Q stage, *n* (%)	
				III	IV
Low MMP-3					
Expression (*n* = 15)	57.00 (52.00–67.00)	6 (40.0)	24.067 ± 2.208	11 (73.3)	4 (26.7)
High MMP-3					
Expression (*n* = 15)	66.00 (49.00–73.00)	13 (86.7)	22.447 ± 2.439	2 (13.3)	13 (86.7)
*p* value*	0.299	**0.021**	0.067	**0.003**	

*BMI* body mass index, *POP-Q* pelvic organ prolapse quantification

*p values from Student’s *t* test, Mann–Whitney *U* test, or Chi-squared test for unequal variances

Significant data (*p* < 0.05) are indicated in bold

Similarly, collagen I expression was classified as high expression (≥ 4 points) and low expression (< 4 points), with a median interpretation result of 4 points. The findings indicated that there were 16 patients with high expression of collagen I and 14 cases with low expression among 30 samples of POP tissue. Notably, low expression of collagen I was significantly associated with menopause (*p* = 0.026) and the severity of POP (*p* = 0.004, Table 5).

**Table 5:** Clinical relevance of collagen I in pelvic organ prolapse

Group	Age (years)	Menopause, *n* (%)	BMI (kg/m^2^)	POP-Q stage, *n* (%)	
				III	IV
Low collagen I expression (*n* = 14)	64.50 (51.75–72.25)	12 (85.7)	22.817 ± 2.721	2 (14.3)	12 (85.7)
High collagen I expression (*n* = 16)	58.50 (49.75–67.00)	7 (43.8)	23.642 ± 2.157	11 (68.8)	5 (31.3)
*p* value*	**0.677**	0.026	0.362	**0.004**	

*BMI* body mass index, *POP-Q* pelvic organ prolapse quantification

*p values from Student’s *t* test, Mann–Whitney *U* test, or Chi-squared test for unequal variances

Significant data (*p* < 0.05) are indicated in bold

Additionally, collagen III expression was classified as high expression (≥ 4 points) and low expression (< 4 points), with a median interpretation result of 4 points. The findings indicated that there were 16 patients with high expression of collagen III and 14 patients with low expression among 30 samples of POP tissue. Notably, low expression of collagen III was significantly associated with menopause (*p* = 0.026) and the severity of POP (*p* = 0.033, Table 6).

**Table 6:** Clinical relevance of collagen III in pelvic organ prolapse

Group	Age (years)	Menopause, *n* (%)	BMI (kg/m^2^)	POP-Q stage, *n* (%)	
				III	IV
Low collagen III expression (*n* = 14)	66.00 (53.00–73.00)	12 (85.7)	23.147 ± 2.656	3 (21.4)	11 (78.6)
High collagen III expression (*n* = 16)	57.00 (49.00–67.00)	7 (43.8)	23.353 ± 2.298	10 (62.5)	6 (37.5)
*p* value*	0.393	0.026**0.026**	0.821	**0.033**	

*BMI* body mass index, *POP-Q* pelvic organ prolapse quantification

*p values from Student’s *t* test, Mann–Whitney *U* test, or Chi-squared test for unequal variances

Significant data (*p* < 0.05) are indicated in bold

## Discussion

The development of POP is a gradual pathological process that can take years or even decades from the onset of symptoms to the evolution of severe POP, which can be distressing for women across all ages [2]. In our study, we observed significantly higher levels of vaginal pH, H_2_O_2_ positivity, and LE positivity in women with POP than in non-POP patients, indicating an association between vaginal microecological dysregulation and the occurrence of POP. Furthermore, mRNA expression levels of DCN, TGF-β1, and MMP-3 were found to be elevated in POP patients whereas collagen I and collagen III expression levels were decreased. The immunohistochemical analysis revealed an upregulation of MMP-3 and a downregulation of collagen I/III in patients with POP, indicating that the high expression of MMP-3 and low expression of collagen I/III are closely associated with menopause and the severity of POP. Abnormal vaginal pH, H_2_O_2_ positivity, and LE positivity correlated positively with MMP-3, whereas H_2_O_2_ positivity correlated negatively with collagen I/III. Furthermore, the high expression of MMP-3 and the low expression of collagen I and collagen III correlated significantly with menopause and the severity of POP. Therefore, we postulate that vaginal microecology may affect the development or progression of POP.

Vaginal microecology is an integral part of human microecology, consisting of vaginal microbiota, endocrine regulatory system, anatomical structure, and local immune system. The vagina harbors a diverse range of microorganisms, with *Lactobacillus* being the predominant bacterium. *Lactobacillus* can suppress the growth of pathogenic bacteria through the secretion of lactic acid, production of H_2_O_2_, and *Lactobacillus* [15, 16]. Previous studies have shown that elevated levels of H_2_O_2_ can induce MMP expression in human pelvic floor fibroblasts, thereby inhibiting collagen expression and promoting POP [17]. Abramov et al. demonstrated that *Lactobacillus crispatus* 2029, isolated from the vagina of healthy reproductive-age females, recognized TLR2/6 receptors and influenced the expression of collagen I and collagen III, ultimately promoting the development of POP [18]. It is suggested that vaginal microecological dysbiosis may activate the local immune system to generate relevant inflammatory response factors, which persistently produce reactive molecules through leukocyte infiltration and destroy surrounding tissue structure and cellular components, ultimately leading to the development of POP [19]. The current study found that abnormal vaginal pH and H_2_O_2_ positivity were significantly higher in the POP group than in the non-POP group, indicating an imbalance of vaginal microecology in patients with POP. This dysregulation may lead to an increase in LE, resulting in inflammation that ultimately contributed to sustained action leading to the development of POP.

Previous studies have demonstrated a close correlation between alterations in collagen ultrastructure and the development of POP [20]. As a vital component of the extracellular matrix supporting pelvic floor stability and plasticity, decreased collagen fiber count leads to relaxation of supporting structures (ligaments, fascia, etc.), ultimately inducing POP. Collagen I/III, the major components of tissues, are synthesized and secreted by fibroblasts. They undergo modifications such as hydroxylation and glycosylation before being degraded by MMPs [13]. Therefore, collagens and MMPs play crucial roles in the pathophysiology of POP. Alperin and Moalli reported that alterations in the metabolism of collagens and elastin in POP, which affected the interactions between different types of fibers in the extracellular matrix and disrupted support and stabilization [21]. Ruiz et al. demonstrated that patients with POP exhibit elevated levels of MMPs and reduced levels of xollagen I in the anterior vaginal wall, indicating that heightened MMP expression and increased collagen degradation impaired fibroblast function within the anterior vaginal tissue, thereby promoting the development of POP [22]. Moreover, Zeng et al. demonstrated reductions in the expression of both collagen I and collagen III within the sacral ligament and vaginal wall tissues of patients with POP [23]. These studies suggested that decreased expression of collagens might compromise the integrity of pelvic floor support structures, ultimately leading to the development of POP. In contrast, Knuuti et al. discovered a significant increase in the concentration of collagen III among patients with POP [24]. In this study, the expression levels of MMP-3 were found to be elevated whereas the levels of collagen I/III were reduced in the POP group. Further stratification revealed that MMP-3 levels were higher in patients with POP-Q stage IV than in those with POP-Q stage III, whereas levels of collagen I/III were lower in POP-Q stage IV patients. Our study confirmed that patients with POP exhibit decreased collagen expression, which may be attributed to the upregulation of MMP-3 expression.

Currently, there is a paucity of studies investigating the pathogenesis of POP. Through sequencing genes related to pelvic floor ligaments, several susceptibility genes in patients with POP were found to be associated with the Wnt classical signaling pathway [25–27]. Wang et al. found that the expression of Wnt 16 and β-catenin was reduced at both gene and protein levels in patients with POP, indicating an inhibition of the classical Wnt signaling pathway in these patients, which may contribute to decreased fibroblast proliferation and collagen secretion, ultimately leading to POP formation [28]. The application of Wnt inhibitors or the inactivation of key protein genes within the Wnt/β-catenin signaling pathway demonstrated significant anti-fibrotic and anti-ECM aggregation effects [27]. The classical Wnt signaling pathway may be inhibited in patients with POP, resulting in reduced fibroblast growth and proliferation activity, decreased secretion of collagen I/III, and elevated MMP-3 levels contributing to increased collagen degradation. This leads to a decrease in the supporting tone of the pelvic floor ligaments, ultimately causing or exacerbating POP. Therefore, we hypothesize that vaginal microecological dysbiosis may result in increased secretion of MMP-3 in uterosacral ligament tissue and subsequent collagen degradation, ultimately leading to decreased collagen content.

There are certain limitations to this study. First, the multifaceted nature of factors influencing POP and the small sample size in this study precluded inclusion of other risk factors such as occupation and surgical technique, etc. Second, no further cytological experiments were conducted in this study, leaving the exact mechanism by which vaginal microecology contributes to POP unclear. Finally, given the retrospective design and small sample size of this study, further prospective multicenter studies are warranted to validate our findings.

## Conclusions

In conclusion, our findings suggest that the levels of vaginal pH, H_2_O_2_ positivity and LE positivity were significantly higher in women with POP than in non-POP patients, indicating vaginal microecological dysbiosis might have an impact on the occurrence of female POP.

## Appendix

Tables 7, 8

**Table 7:** Characteristics of patients with pelvic organ prolapse (*POP*) compared with those with non-pelvic organ prolapse

Variable	POP group (*n* = 30)	Non-POP group (*n* = 30)	*p* value
Age (years)	63.50 (51.25,69.75)	59.50 (52.00,64.25)	0.297
BMI (kg/m^2^)	23.257 ± 2.430	22.544 ± 1.677	0.192
Reproductive history, *n* (%)			0.286
≤ 1	11 (36.7)	16 (53.3)	
2–4	14 (46.7)	10 (33.3)	
≥ 5	5 (16.8)	4 (13.4)	
Diabetes, *n* (%)			0.519
No	23 (76.7)	25 (83.3)	
Yes	7 (23.3%)	5 (16.7)	
Hypertension, *n* (%)			0.385
No	26 (86.7)	28 (93.3)	
Yes	4 (13.3)	2 (6.7)	
Total	30	30	

*BMI* body mass index

**p* values from Student’s *t* test, Mann–Whitney *U* test, or Chi-squared test for unequal variances

**Table 8:** Characteristics in patients with pelvic organ prolapse quantification (*POP-Q*) stage III compared with those of POP-Q stage IV patients

Variable	POP-Q stage III (*n* = 13)	POP-Q stage IV (*n* = 17)	*p* value*
Age (years)	54.00 (49.00, 67.00)	65.00 (55.00, 73.50)	0.106
BMI (kg/m^2^)	23.146 ± 2.062	23.341 ± 2.737	0.831
Reproductive history, *n* (%)			0.153
≤ 1	3 (23.1)	8 (47.1)	
2–4	6 (46.1)	8 (47.1)	
≥ 5	4 (30.8)	1 (5.8)	
Diabetes, *n* (%)			0.666
No	9 (69.2)	14 (82.4)	
Yes	4 (30.8)	3 (17.6)	
Hypertension, *n* (%)			1.000
No	11 (84.6)	15 (88.2)	
Yes	2 (15.4)	2 (11.8)	
Total	13	17	

*BMI* body mass index, *POP* pelvic organ prolapse

^*^*p* values from Student’s *t* test, Mann–Whitney *U* test, or Chi-squared test for unequal variances

## Abbreviations

DCN: Decorin
H_2_O_2_: Hydrogen peroxide
MMP-3: Matrix metalloproteinase-3
POP: Pelvic organ prolapse
